# Antibodies against small heat-shock proteins in Alzheimer’s disease as a part of natural human immune repertoire or activation of humoral response?

**DOI:** 10.1007/s00702-015-1477-2

**Published:** 2015-11-14

**Authors:** Ewa Papuć, Witold Krupski, Ewa Kurys-Denis, Konrad Rejdak

**Affiliations:** Department of Neurology, Medical University of Lublin, 8 Jaczewskiego Str., 20-954 Lublin, Poland; Second Department of Radiology, Medical University of Lublin, Lublin, Poland

**Keywords:** Alzheimer disease, Small heat-shock proteins, HSP60, Alpha B-crystallin, Autoantibodies, Humoral response, Immune system

## Abstract

Characterization of autoantibodies specific for some disease-related proteins, would allow to better assess their role as diagnostic and prognostic markers. In the light of increasing evidence for both humoral and cellular adaptive immune responses in the pathophysiology of Alzheimer’s disease (AD), and data on the increased small heat-shock proteins (sHSP) expression in this disease, it seemed justified to assess humoral response against sHSP in AD patients. The aim of the study was to check whether AD has the ability to elicit immune response against small HSP, which could also serve as disease biomarkers. IgG and IgM autoantibodies against alpha B-crystallin and anti-HSP 60 IgG autoantibodies were assessed in 59 AD patients and 59 healthy subjects. Both IgM and IgG autoantibodies against alpha B-crystallin in AD patients were significantly higher compared to healthy controls (*p* < 0.05). No statistically significant differences were found between AD patients and healthy subjects were found in anti-HSP60 IgG autoantibody titers (*p* = 0.29). Anti-HSP60 antibodies present in AD patients may indeed belong to natural human immune repertoire, and chronic neurodegenerative process does not have significant inducing effect on the systemic immunoreactivity against HSP60. Increased titers of IgM and IgG autoantibodies against alpha B-crystallin in AD patients may reflect activation of humoral immune response in the course of this chronic disease, probably secondary to its increased expression. Further prospective studies, on larger group of AD patients and measuring a change in antibodies titers with disease progression are necessary to assess the exact role of these antibodies in AD.

## Introduction

Identification of disease-specific diagnostic and prognostic biomarkers which would allow for an early detection and clinical follow-up of Alzheimer’s disease (AD) patients is very important. As there is increasing evidence for both humoral and cellular adaptive immune responses in the pathophysiology of AD, assessment of disease-related B and T cell responses may constitute a promising source of potential early biomarkers specific for certain disorders. Characterization of autoantibodies specific for some disease-related proteins, would allow to better decipher their role as early diagnostic and prognostic markers in AD. In the light of previously emerging hypotheses on the activation of the adaptive immune system against amyloid β (Aβ) aiming to decrease the accumulation of this peptide in the brain (Solomon et al. 1997; Schenk et al. 1999), it seemed justified to assess humoral response against other peptides which are overexpressed in AD.

Heat-shock proteins (HSPs) are functionally and immunologically highly conserved molecules present in almost all living organisms (Ellis 2007). HSPs are up-regulated in response to cellular stress to protect the cell from a variety of stresses (Kelly and Yenari 2002). This increased HSP expression takes place in cells exposed to mild stress and protects them against subsequent stress. However, in cells subjected to severe stress, HSP promote apoptosis.

In AD, HSPs expression is associated with deposition of Aβ and neurofibrillary tangles, and recent findings suggest that HSPs prevent the accumulation of Aβ (Abdul et al. 2006; Evans et al. 2006; Shimura et al. 2004).

In the light of evidence for increased expression of some HSPs in brain tissues in patients with AD (Björkdahl et al. 2008) and also in brains of patients with mild cognitive impairment (MCI) (Di Domenico et al. 2010), we decided to assess humoral response against sHSP in AD, and to look for potential biomarkers of the disease.

To test this hypothesis, we assessed the presence of autoantibodies against small HSP, like alpha B-crystallin and HSP 60 in sera of patients suffering from AD. We assessed all measurements from AD patients in relation to autoantibody levels in healthy control subjects.

### Alpha B-crystallin

Alpha B-crystallin is a small heat-shock protein (sHSP), which occurs at increased levels in brains of Alzheimer’s disease patients, and co-localizes with amyloid β (Aβ) (Renkawek et al. 1994). AD pathology involves not only aggregation of abnormal proteins, but also their decreased degradation, and cytoskeletal disruption. Small HSPs take part in protein degradation and protection against protein aggregation, and they interact with several cytoskeletal components such as microtubules (MT) and neurofilaments (NF). There is evidence that some small heat-shock proteins (sHSPs), like Hsp27 and alpha B-crystallin, are up-regulated in AD, especially in the regions commonly affected by AD but its consequences are still largely unknown (Björkdahl et al. 2008; Mao et al. 2001). The presence and increased sHsps expression in AD brains may indeed reflect a defensive response to prevent amyloid fibril formation and its toxicity (Renkawek and Bosman 1995).

### HSP 60 and related autoantibodies

Anti-60 kD heat-shock protein (Hsp60) antibodies are present in sera of healthy human subjects (Varbiro et al. 2010), also in sera of patients with inflammatory and autoimmune disorders (Yokota and Fujii 2010; Mayr et al. 1999). In the light of evidence for inflammatory process present in human brains of patients with AD, as well as data on increased expression of different sHSP in neurodegenerative disorders, it may be hypothesized that AD may be also accompanied by the presence of anti-HSP antibodies.

Thus, we decided to assess the humoral response against sHSP in sera of AD patients in comparison to healthy controls, to assess the presence of adaptive immune response in AD and to look for early biomarkers of the disease.

## Materials and methods

59 AD patients in different clinical stages of the disease [on Clinical Dementia Rating (CDR) grade 0.5–3] treated in the Department of Neurology of Medical University of Lublin, Poland were enrolled. All AD patients fulfilled NINCDS–ADRDA criteria for probable Alzheimer’s disease diagnosis (McKhann et al. 1984). AD patients were divided into three subgroups: mild AD (CDR 0.5–1), moderate (CDR 2) and severe (CDR 3).

Serum samples from all 59 AD patients were examined for the presence of IgG and IgM autoantibodies against alpha B-crystallin and IgG antibodies against HSP 60. In addition, serum samples from 59 healthy controls matched for age and gender were assessed for the same antibodies.

IgG and IgM autoantibodies against alpha B-crystallin were measured by a commercially available ELISA system according to the instructions of the manufacturer (Mediagnost, Germany). All analysis were performed in duplicate. The ELISA (E100) uses an internal standard pool serum for calculation of antibody titers and employing microplates coated with myelin-specific proteins purified from bovine brain. The autoantibody titer was calculated after the subtraction of nonspecific binding and blanks. The titers were estimated on the base of calibration curve of autoantibody standards and expressed in Mediagnost Units per milliliter (MU/mL).

For measurement of anti-HSP 60 antibodies we used enzyme-linked immunosorbent assay, which is a validated method (ADI-EKS-650, Enzo Life Sciences). Concentration values were expressed in ng/mL. Assay Designs Anti-Human Hsp60 (total) ELISA Kit uses recombinant human Hsp60 bound to the wells of the immunoassay plate to bind anti-human Hsp60 antibodies present in human serum.

Blood samples were collected between 8:00 and 10:00 a.m., transferred to the lab on ice, centrifuged and serum was stored at −70 °C within 60 min thereafter. The study was approved by the local Ethics Committee of Medical University of Lublin, Poland, and all study participants gave written informed consent for study participation.

### Statistical analysis

Antibodies titer differences between study group and the control subjects were estimated with the usage of ANOVA test. For AD subgroups analysis Kruskal–Wallis and *U* Mann–Whitney tests were applied. *p* value <0.05 was considered statistically significant (two sided). Statistical calculations were done with the usage of InStat GraphPad Software Inc, CA.

## Results

### Antibodies against alpha B-crystallin

We confirmed the presence of IgM and IgG autoantibodies against alpha B-crystallin in investigated groups of AD patients and healthy subjects, and observed statistically significant higher levels of both IgG (*p* < 0.05) and IgM autoantibodies (*p* < 0.05) titers in AD patients compared to healthy control subjects. In AD subgroup analysis, we have found statistically significant higher levels of IgG antibodies titers in patients with severe AD, as compared to patients with mild disease severity (*p* = 0.003). In comparison of AD subgroups with very mild and mild dementia (CDR 0.5–1.0) versus moderate and severe (CDR 2–3), we also confirmed significantly higher IgGs antibodies titers for more advanced AD patients (*p* = 0.03). No differences were observed in IgM titers among subgroups of AD patients.

### Anti-HSP60 autoantibodies

We have not observed statistically significant differences in levels of anti-HSP 60 IgG autoantibodies between AD patients and healthy controls (*p* > 0.05). Also in the AD subgroup analysis we have not found statistically significant differences in the anti HSP60 antibodies titers between investigated subgroups.

Demographical, clinical and biochemical characteristics of the study population are shown in Tables 1, 2 and 3.

**Table 1:** Demographic and clinical data of the study population

Demographic characteristic of study group	Alzheimer’s disease patients	Control subjects
Subjects (female/male)	59 (26/33)	59 (29/30)
Age (years) ± SD (range)	72.93 ± 7.36 (55–84)	72.58 ± 5.86 (58–82)
Disease duration (years) ± SD (range)	7.65 ± 2.69 (4–13)	NA
MMSE (0–30) ± SD (range)	16.86 ± 4.45 (9–24)	28.54 ± 1.07 (27–30)
CDR ± SD (range)	2.16 ± 0.74 (0.5–3)	NA

Data are presented as means with standard deviation (SD)

*MMSE* mini mental state examination, *CDR* clinical dementia rating, *NA* not applicable

**Table 2:** Biochemical data of the study population

Biochemical data	Alzheimer’s disease patients (*n* = 59)	Control subjects (*n* = 59)	ANOVA, *p* value
Anti-alpha B-crystallin antibodies			
IgG titer (MU/mL), mean ± SD (range)	2.86 ± 0.94 (1.07–4.95)	1.10 ± 0.73 (0.15–3.3)	*p* < 0.05
IgM titer (MU/mL), mean ± SD (range)	11.83 ± 2.31 (7.59–16.99)	6.14 ± 1.49 (4.2–9.4)	*p* < 0.05
Anti-HSP60 antibodies			
IgG titer (MU/mL), mean ± SD (range)	1.95 ± 0.76 (0.69–3.67)	1.79 ± 0.88 (0.76–5.82)	*p* = 0.29

Data are presented as means with standard deviation (SD) and range. *p* < 0.05, significant difference in comparison to control

**Table 3:**
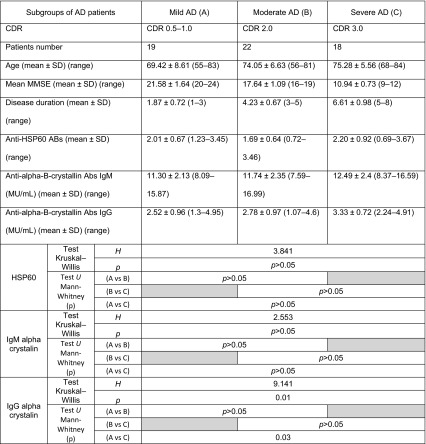
Demographic clinical and biochemical characteristic of subgroups of Alzheimer’s disease patients

Data are presented as means with standard deviation (SD)

*CDR* Clinical Dementia Rating Scale, *MMSE* mini mental state examination, *AD* Alzheimer’s disease, *Abs* antibodies

## Discussion

### Autoantibodies against alpha B-crystallin

Here we provided evidence for the presence of humoral response against sHSPs in AD. Alpha B-crystallin suppresses the aggregation and precipitation of a wide range of proteins, including formation of amyloid fibrils in AD. Data on the increased expression of sHSP in Alzheimer’s disease have been confirmed in many studies (Björkdahl et al. 2008; Renkawek et al. 1994; Shinohara et al. 1993). Higher IgG and IgM antibodies titers against alpha B-crystallin in AD patients, confirmed in this study, may reflect the increasing role of this protein in prevention of Aβ fibril formation in the course of neurodegenerative process, and is probably secondary to increased expression of this protein. This hypothesis may be additionally supported by the increase of the IgG antibodies titers with more advanced neurodegenerative process, which we confirmed in the study.

However, it is not completely clear whether found antibodies against sHSP reflect diffuse CNS injury or contribute to this injury. Monahan et al. (2008) presented the model on how the immune adaptive response may be involved in the pathogenesis and progression of neurodegenerative disorders. Progressive neurodegenerative disorder is accompanied by death of the CNS cells and presentation of their new antigens to the immune system, with subsequent activation of T and B cells. B cells or specific autoantibodies may then enter the CNS across dysfunctional blood–brain barrier (BBB), produce cytokines which activate microglia, and release autoantibodies. This may lead to further inflammation and subsequent cellular death (Monahan et al. 2008). Recent data suggest blood–brain barrier dysfunction in the course of neurodegenerative process (Carvey et al. 2009; Viggars et al. 2011; Simpson et al. 2010).

It should be mentioned here, however, that there is evidence on the role of alpha B-crystallin, not as a target for the autoimmune response, but rather as a chaperone, which helps to bind different antibodies irrespective of their specificity (Rothbard et al. 2001). Rothbard et al. (2001) in their study, performed on multiple sclerosis (MS) patients, showed that small HSPs (like alpha B-crystallin) bind different immunoglobulins (Igs) with high affinity, and in fact are receptors of the Igs, not the antigens for them. This would mean that alpha B-crystallin does not elicit itself specific immune response (Rothbard et al. 2001). Comparable immunoreactivity to alpha B-crystallin of sera of MS patients and healthy controls was previously described by van Noort (2006). For this reason, the assessment of humoral response in different inflammatory disorders may be difficult, as majority of immunoassays are based on typical antibody–antigen interaction, and they do not consider the possibility of the antigen binding the antibody, and this can be also the limitation of our study. Whether results presented by Rothbard (2001) are disease specific or can be adopted to different other inflammatory disorders, requires further investigations. Contrary to their study, we observed significantly higher immunoreactivity to alpha B-crystallin in sera of AD patients compared to healthy subjects.

### Autoantibodies against HSP 60

The study confirmed the presence of anti-HSP 60 autoantibodies in sera of AD patients and healthy subjects, without statistically significant differences between investigated subgroups. AD patients presented, however, slightly increased antibodies titers compared to healthy subjects. This is interesting in the light of our recent study (Papuć et al. 2015), where we confirmed significantly increased anti-HSP 60 autoantibodies titers in another neurodegenerative disorder, Parkinson’s disease.

There is evidence that in AD both peripheral and brain endogenous inflammatory processes enhance the disease progression (Monsonego et al. 2013). Additionally, a growing body of evidence demonstrates that Aβ plaques induce an inflammatory reaction in the brain (McGeer et al. 2005; Vom Berg et al. 2012). Recent studies demonstrated the significant pathological effect of Aβ on cerebral amyloid angiopathy that causes vascular inflammation, brain hemorrhages, compromised perivascular drainage and altered blood flow (Meyer et al. 2008; Thal et al. 2008).

Inflammatory processes such as microglia, astrocytes and complement activation, cytokine elevation and acute phase protein changes are thought to represent, at least partially, a response to the accumulation of Aβ in the vasculature and parenchyma of the brain. A compromised immune system may have substantial impact on these processes and lead to neuronal repair processes, which enhance the progression of AD.

We hypothesized that in the light of evidence for increased expression of sHSP in different neurodegenerative disorders (Björkdahl et al. 2008), and evidence of activation of humoral immunoreactivity to other protein depositions, like Aβ (Monsonego et al. 2013), one can expect activation of humoral response against this HSP60, as we previously confirmed for Parkinson’s disease (Papuć et al. 2015). In this study, we confirmed comparable immunoreactivity against HSP 60 in AD and healthy subjects, with no influence of AD progression on anti-HSP60 antibodies titers.

The presence of anti-HSP 60 autoantibodies is healthy people and in different disorders is still unclear. They could be cross-reacting antibodies induced by bacterial infections (Mayr et al. 1999) or real autoantibodies (Cohen and Young 1991). Numerous data support that the carriage of anti-Hsp60 autoantibodies may be a part of natural antibody repertoire, which can be an inherited trait, and the cumulative antibody-inducing effects of multiple infections add to this trait (Varbiro et al. 2010; Zlacka et al. 2006). Natural antibodies refer to antibodies that are present in the serum of healthy individuals without overt immunization or infection (Coutinho et al. 1995). Thus, the presence of anti-self Hsp autoantibodies may be an integral part of the normal immune function, playing role in self-protection and regulation of autoimmunity. In humans natural autoantibodies may belong to IgG, IgM and IgA isotypes with the predominance of IgG (Lacroix-Desmazes et al. 1995).

The presence of anti-HSP 60 antibodies, as a part of natural human immune repertoire, has been confirmed in different other disorders, as well as in healthy subjects (Varbiro et al. 2010; Lacroix-Desmazes et al. 1995; Zlacka et al. 2006).

**Fig. 1:**
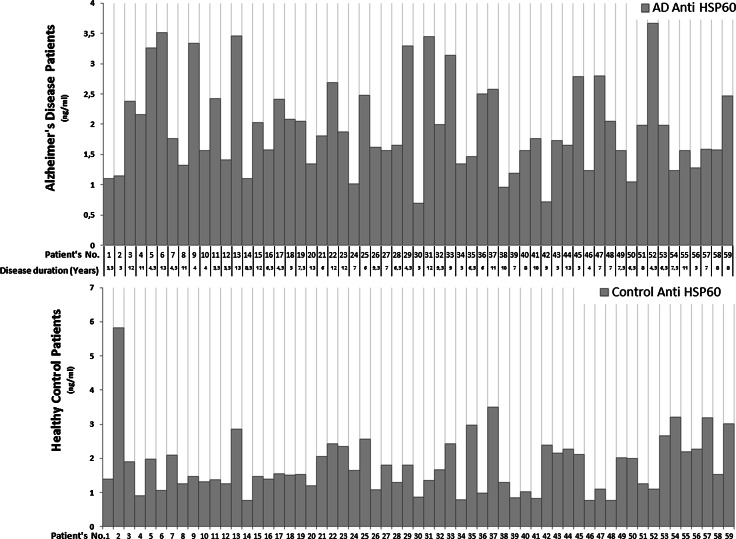
Variability of anti-HSP60 antibodies titers in investigated groups of patients. Alzheimer’s disease patients and healthy control subjects

Although natural autoantibodies levels against different conserved proteins remain stable with time, large inter-individual differences may be observed (Mouthon et al. 1996), nevertheless, the autoreactive repertoires are highly conserved among individuals (Varbiro et al. 2010; Mouthon et al. 1995). This unique reactivity pattern very characteristic of each individual is described as an “antibody immuno-fingerprinting”. Our results are in accordance with data from previously performed studies, as we also observed large variability of anti-HSP60 IgG antibodies titers, which is presented in Fig. 1.

Of course, we cannot exclude the influence of factors other than chronic neurodegenerative process on the presence of discussed immune response against HSP60. HSP are considered one of the superantigens and are the immunodominant antigens of various microbial pathogens inducing strong humoral and cellular immune responses in numerous infections caused by bacteria, protozoa, fungi and nematodes (Shinnick 1991; Kaufmann and Schoel 1994). Recent data suggest that particularly the viral infections may influence the activity of sHSP and subsequent autoimmunity (Temajo and Howard 2004; Rajaiah and Moudgil 2009).

Based on results of our study, we admit that anti-HSP60 antibodies may belong to natural autoantibodies repertoire, and chronic neurodegenerative process probably has additional, but rather poor influence on activation of the immune system against HSP60.

## Conclusions

Anti-HSP60 antibodies present in AD patients may indeed belong to natural human immune repertoire, and chronic neurodegenerative process does not have significant inducing effect on the systemic immunoreactivity against HSP60.

Increased titers of IgM and IgG autoantibodies against alpha B-crystallin in AD patients may reflect activation of humoral immune response in the course of this chronic disease, probably secondary to increased expression of this heat-shock protein. It is still unclear whether the examined autoantibodies are primary factors responsible for neurodegeneration, secondary phenomenon which occurs in response to widespread neurodegenerative process, or belong to natural human immune repertoire, without having any pathogenic role. Further prospective studies on larger group of AD patients and measuring a change in antibodies titers with disease progression are necessary to assess the exact role of these antibodies in AD.
